# Early pulmonary events of nose-only water pipe (shisha) smoking exposure in mice

**DOI:** 10.14814/phy2.12258

**Published:** 2015-03-16

**Authors:** Abderrahim Nemmar, Ahmed Al Hemeiri, Naser Al Hammadi, Priya Yuvaraju, Sumaya Beegam, Javed Yasin, Mohamed Elwasila, Badreldin H Ali, Ernest Adeghate

**Affiliations:** 1 Department of Physiology, College of Medicine and Health Sciences, United Arab Emirates UniversityAl Ain, United Arab Emirates; 2 Department of Internal Medicine, College of Medicine and Health Sciences, United Arab Emirates UniversityAl Ain, United Arab Emirates; 3 Department of Pharmacology, College of Medicine and Health Sciences, United Arab Emirates UniversityAl Ain, United Arab Emirates; 4 Department of Pharmacology and Clinical Pharmacy, College of Medicine & Health Sciences, Sultan Qaboos UniversityAl-Khod, Sultanate of Oman; 5 Department of Anatomy, College of Medicine and Health Sciences, United Arab Emirates UniversityAl Ain, United Arab Emirates

**Keywords:** Airway resistance, inflammation, nose-only exposure, oxidative stress, water pipe smoking

## Abstract

Water pipe smoking (WPS) is increasing in popularity and prevalence worldwide. Convincing data suggest that the toxicants in WPS are similar to that of cigarette smoke. However, the underlying pathophysiologic mechanisms related to the early pulmonary events of WPS exposure are not understood. Here, we evaluated the early pulmonary events of nose-only exposure to mainstream WPS generated by commercially available honey flavored “*moasel*” tobacco. BALB/c mice were exposed to WPS 30 min/day for 5 days. Control mice were exposed using the same protocol to atmospheric air only. We measured airway resistance using forced oscillation technique, and pulmonary inflammation was evaluated histopathologically and by biochemical analysis of bronchoalveolar lavage (BAL) fluid and lung tissue. Lung oxidative stress was evaluated biochemically by measuring the level of reactive oxygen species (ROS), lipid peroxidation (LPO), reduced glutathione (GSH), catalase, and superoxide dismutase (SOD). Mice exposed to WPS showed a significant increase in the number of neutrophils (*P* < 0.05) and lymphocytes (*P* < 0.001). Moreover, total protein (*P* < 0.05), lactate dehydrogenase (*P* < 0.005), and endothelin (*P* < 0.05) levels were augmented in bronchoalveolar lavage fluid. Tumor necrosis factor α (*P* < 0.005) and interleukin 6 (*P* < 0.05) concentrations were significantly increased in lung following the exposure to WPS. Both ROS (*P* < 0.05) and LPO (*P* < 0.005) in lung tissue were significantly increased, whereas the level and activity of antioxidants including GSH (*P* < 0.0001), catalase (*P* < 0.005), and SOD (*P* < 0.0001) were significantly decreased after WPS exposure, indicating the occurrence of oxidative stress. In contrast, airway resistance was not increased in WPS exposure. We conclude that subacute, nose-only exposure to WPS causes lung inflammation and oxidative stress without affecting pulmonary function suggesting that inflammation and oxidative stress are early markers of WPS exposure that precede airway dysfunction. Our data provide information on the initial steps involved in the respiratory effects of WPS, which constitute the underlying causal chain of reactions leading to the long-term effects of WPS.

## Introduction

Water pipe smoke (WPS; also called hubble bubble, shisha, hookah, or narghile) is a traditional method of tobacco smoking commonly practiced especially in the Arabian Peninsula, Turkey, India, and China and is now increasing globally (Maziak et al. 2004; Knishkowy and Amitai 2005; Chaouachi 2009; Mandil et al. 2010). For example, in European countries, there has been an increase of WPS use in the past several years (Chaouachi 2009), and in the United States, there are about 300 WPS bars and cafés, located in two-thirds of the states, usually near large colleges and universities (Chan and Murin 2011). WP smokers can smoke from 30 min to a few hours and inhale more deeply because of the less irritating nature of the moisturized smoke. It is wrongly believed that WPS is much less toxic than cigarettes. A recent analysis of toxicant yields during 1–2 h WPS was comparable to 100–200 cigarettes (World Health Organization 2005; Neergaard et al. 2007). There is evidence that those who are occasional or regular water pipe smokers are more likely to become regular cigarette smokers, suggesting that WPS may be a potential entryway for regular cigarette use (Jensen et al. 2010).

While there is a large amount of data on the short- and long-term effects of cigarette smoke (CS), there is a paucity of data regarding WPS. A study has reported a higher proportion of chronic bronchitis in WP smokers compared with cigarette smokers and a quasi-permanent alteration in maximum mid-expiratory flow (MMEF 25–75%) in WP smokers compared with cigarette smokers (Mohammad et al. 2008). Forced expiratory volume in 1 sec (FEV1) was more altered in cigarette smokers than in WP smokers (Mohammad et al. 2008). Also, cross-sectional studies reported a significant decrease in the FEV_1_ in the WPS group compared with nonsmokers (Raad et al. 2011). Moreover, it has been reported that one session of WPS resulted in significant increases in carboxyhemoglobin (COHb) concentrations, systolic, and diastolic blood pressures, and heart and respiratory rates (Hakim et al. 2011). A decrease was observed in peak expiratory flow rate (PEFR), forced expiratory flow between 25% and 75% of forced vital capacity (FEF_25–75%_; Hakim et al. 2011). The same research group has more recently shown that one session of active indoor group WPS resulted in significant increases in COHb and serum nicotine levels (8-fold and 18-fold, respectively) and was associated with adverse cardiorespiratory health effects (Bentur et al. 2014).

Clinical studies have reported difficulties in studying the isolated effects of WPS because most of the smokers are also current or past cigarette smokers. Therefore, experimental studies assessing the respiratory adverse effects of WPS are required to shed light on the mechanisms underlying the early pulmonary effects of WPS.

Using whole-body exposure system, it has been recently reported that exposure for 7 days to WPS and cigarette smoke causes pulmonary toxicity (Khabour et al. 2012). The nose-only and whole-body exposure systems are the two main methods presently applied to investigate the effect of tobacco smoke exposure in mice (Wright et al. 2008; Stevenson and Birrell 2011; Rinaldi et al. 2012). The shortcoming of using whole-body exposure is that the animals may ingest nicotine or tar substances when cleaning their fur (Rinaldi et al. 2012). The nose-only exposure system circumvents this problem and most likely best resembles the human situation (Tuder 2006; Tuder et al. 2006; Rinaldi et al. 2012). Yet, only limited studies have used nose-only exposure systems to study the pulmonary effect of tobacco smoke (Nemmar et al. 2012a, 2013c,d; Rinaldi et al. 2012). We have recently demonstrated that exposure to WPS for 1 month increased airway resistance, inflammation, and oxidative stress (Nemmar et al. 2013c). However, the early effects of nose-only WPS exposure on lung function, inflammation, and oxidative stress have not been studied before. One of the reasons why it is important to study this effect is because repetitive subacute effects of WPS may constitute the underlying causal chain of events leading to the eventual long-term effects.

The aim of this study, therefore, was to investigate, in mice, the early pulmonary events (5-days) of nose-only WPS exposure on a comprehensive set of respiratory endpoints comprising pulmonary inflammation and oxidative stress, and airway resistance.

## Material and Methods

### Animals and treatments

This project was reviewed and approved by the Institutional Review Board of the United Arab Emirates University, College of Medicine and Health Sciences, and experiments were performed in accordance with protocols approved by the Institutional Animal Care and Research Advisory Committee.

### WPS exposure

Both male and female BALB/C mice (Taconic Farms Inc., Germantown, NY) weighing 20 ± 2 g were housed in a conventional animal house and maintained on a 12-h light–dark cycle. The animals were placed in cages and supplied with pelleted food and water ad libitum. Following 1 week of acclimatization, animals were randomly divided into air (control) and WPS-exposed groups. Mice were placed in soft restraints and connected to the exposure tower (Nemmar et al. 2012a, 2013b,c,d; Rinaldi et al. 2012). They were exposed to WPS or air through their noses using a nose-only exposure system (InExpose System; SCIREQ, Montreal, QC, Canada). Animals were exposed to mainstream WPS generated by commercially available honey flavored “*moasel*” tobacco (Al Fakher, Ajman, UAE). The tobacco was lit with instant light charcoal disk (Star, 3.5 cm diameter and 1 cm width). A standard of one puff of 2-sec duration was taken once a minute, followed by 58 sec of fresh air at a rate of 6 mL/sec was applied. The duration of the session was 30 min/day for 5 days. The 30-min duration of the exposure session was selected from a recently published study that has assessed the cardiorespiratory effects of WPS in human subjects (Hakim et al. 2011; Bentur et al. 2014). Following the last exposure session (i.e., day 5), lung inflammation and oxidative stress, and airway resistance were evaluated.

### Collection and analysis of BAL fluid

The collection and analysis of bronchoalveolar lavage (BAL) has been performed according to a previously described method (Hardy et al. 2009; Nemmar et al. 2009, 2011, 2012b). In brief, in separate animal groups (*n* = 8 per group), mice were sacrificed with an overdose of sodium pentobarbital after WPS or air exposure. The trachea was cannulated and lungs were lavaged three times with 0.7 mL (a total volume of 2.1 mL) of sterile NaCl 0.9% solution. The recovered fluid aliquots were pooled. No difference in the volume of collected fluid was observed between the different groups. BAL fluid was centrifuged (for 10 min at 1000 ×* g*, 4°C). The supernatant was stored at −80°C until further analysis for total proteins (kit from Biorad, Munich, Germany), endothelin (kit from Cayman Chemical, Ann Arbor, MI), and lactate dehydrogenase (LDH) analysis by UV assay using a commercially available kit (Roche, Basel, Switzerland) in blood chemistry analyzer (Roche COBAS).

### Histology

The right lung of the same animals used for BAL was fixed with 10% formaldehyde after the lavage (Vanoirbeek et al. 2004). Seven-micrometer sections were prepared from the paraffin blocks of right lung and stained with hematoxylin and eosin. A histologist examined slices from different lung lobes and evaluated without prior knowledge of the animals' treatment. The degree of interstitial infiltration by inflammatory cells, in response to WPS exposure, was evaluated by counting the number of inflammatory cells in the lung at high power field (×100) as previously described (Nemmar et al. 2012a).

### Measurement of the levels of reactive oxygen species (ROS), lipid peroxidation (LPO), reduced glutathione (GSH), catalase, and superoxide dismutase (SOD) in lung tissue

In separate mice (*n* = 8 in each group), following the exposure to WPS or air, animals were sacrificed by an overdose of sodium pentobarbital. Lung tissues from air-exposed control and WPS-exposed mice were collected and rinsed with ice-cold PBS (pH 7.4) before homogenization in 0.1 mol/L phosphate buffer pH 7.4 containing 0.15 mol/L KCl, 0.1 mmol/L EDTA and 0.1 mmol/L phenylmethylsulfonylfluoride at 4°C. Homogenates were centrifuged for 10 min at 3000 × *g* at 4°C to remove cellular debris, and supernatants were used for further analysis. Protein content was measured by Bradford's method as described before (Nemmar et al. 2012a).

Reactive oxygen species were measured in whole-lung tissue homogenates, which were obtained as described above, using 2′,7′-dichlorofluorescein diacetate (Molecular Probes, Eugene, OR) as a fluorescent probe as described previously (LeBel et al. 1990; Nemmar et al. 2013c). The results were normalized as ROS produced per milligram of protein.

NADPH-dependent membrane lipid peroxidation was measured as thiobarbituric acid reactive substance using malonedialdehyde as standard (Sigma–Aldrich Fine Chemicals, St Louis, MO; Nemmar et al. 2012a).

Measurement of GSH concentrations was carried out in control and WPS-exposed animals according to the method described by commercially available kit (Sigma–Aldrich Fine Chemicals, Munich, Germany).

Measurement of SOD and catalase activities were carried out in control and WPS-exposed animals using spectrophotometric method with commercially available kits (Cayman Chemical).

### Measurement of TNFα and IL-6 in lung tissue

At the end of the exposure period to either WPS or air, animals were sacrificed by an overdose of sodium pentobarbital, and their lungs were quickly collected and rinsed with ice-cold PBS (pH 7.4) before homogenization in 50 mmol/L Tris buffer pH 7.4 containing 400 mmol/L NaCl and 0.5% Triton X-100 at 4°C. The homogenates were centrifuged for 10 min at 3000 × *g* to remove cellular debris, and the supernatants were used for further analysis (Nemmar et al. 2013c,d). Protein content was measured by Bradford's method as described before. The concentrations of IL-6 and TNF α in the lung were determined using ELISA Kits (Duo Set; R & D systems, Minneapolis, MN).

### Airway reactivity to methacholine

In separate group of mice (*n* = 8 in each group), airway hyperreactivity responses were measured using a forced oscillation technique (FlexiVent; SCIREQ). Airway resistance (*R*) was assessed after increasing exposures to methacholine. Mice were anesthetized with an intraperitoneal injection of pentobarbital (70 mg/kg). The trachea was exposed and an 18-gauge metal needle was inserted into the trachea. Mice were connected to a computer-controlled small animal ventilator and quasi-sinusoidally ventilated with a tidal volume of 10 mL/kg at a frequency of 150 breaths/min and a positive end-expiratory pressure of 2 cm H_2_O to achieve a mean lung volume close to that observed during spontaneous breathing. After measurement of a baseline, each mouse was challenged with methacholine aerosol, generated with an in-line nebulizer and administered directly through the ventilator for 5 sec, with increasing concentrations (0, 0.625, 1.25, 2.5, and 5 mg/mL). *R* was measured using a “snapshot” protocol each 20 sec for 2 min. The mean of these five values was used for each methacholine concentration, unless the coefficient of determination of a measurement was smaller than 0.95. For each mouse, *R* was plotted against methacholine concentration (from 0 to 5 mg/mL; Nemmar et al. 2012b; Nemmar et al. 2013a).

### Statistics

All statistical analyses were performed with GraphPad Prism Software version 5 (San Diego, CA, USA). To determine whether parameters were normally distributed, the KS normality test was applied. Normally distributed data were analyzed using the unpaired *t*-test for differences between groups. Nonnormally distributed data (total proteins and LDH in BAL fluid and ROS in lung) were analyzed using Mann–Whitney test for differences between groups. All the data in figures were reported as mean ± SEM. *P* values < 0.05 are considered significant.

## Results

### Lung histology and morphometry

**Figure 1:**
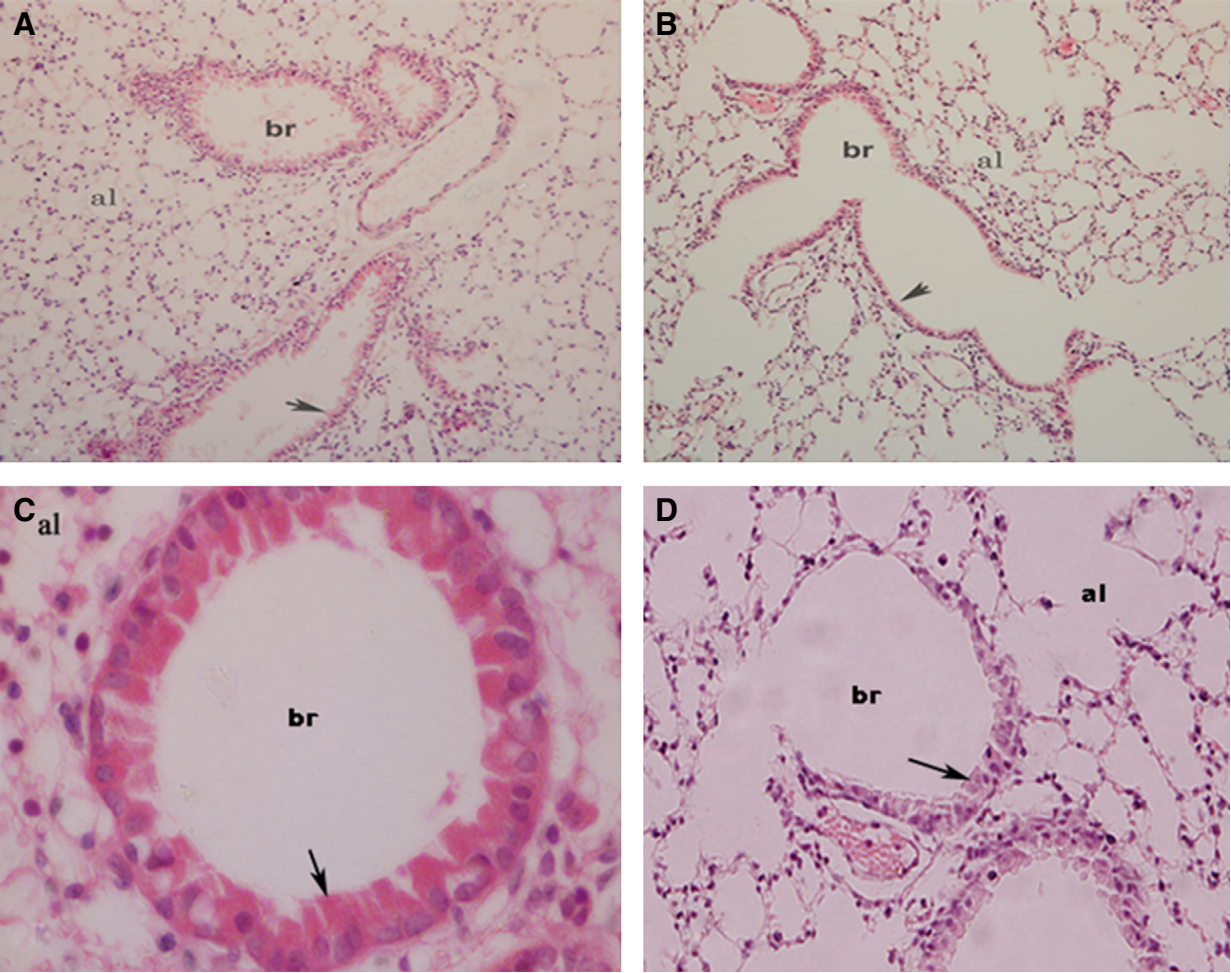
Representative light micrographs of lung tissues of mice following subacute (5 days) nose-only air (control, A and C) or water pipe smoke (WPS, B and D) exposure. Note that the bronchiolar (br) epithelium of the lung of mice exposed to WPS is (1.5 fold) shorter compared with control (short arrows). al, alveolus. Magnification: A and B: ×200; C and D: ×1000.

Sections of lung from air-exposed mice showed a normal appearance under light microscopy (Fig.1A and C). Compared with air-exposed group, the bronchiolar epithelium was shorter in lung sections obtained from mice exposed for 5 days to WPS (Fig.1B and D).

**Figure 2:**
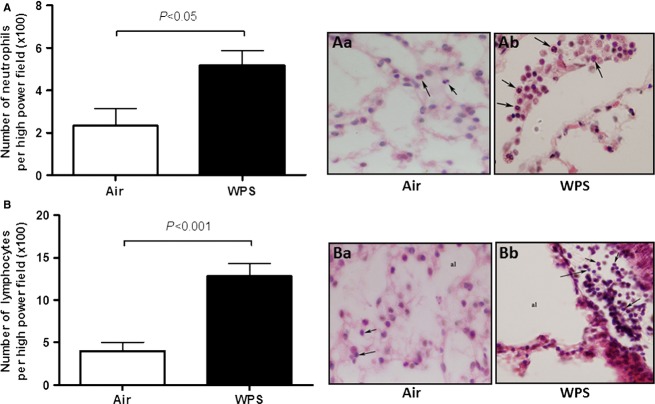
Morphometric analysis showing the number of neutrophils (A) and lymphocytes (B) following subacute (5 days) exposure to air (control) or water pipe smoking (WPS). Aa and Ab show neutrophils (arrows) in representative micrographs of nose-only air (control, Aa) or water pipe smoke (WPS, Ab) exposure, respectively. Ba and Bb show lymphocytes (arrows) in the light micrographs of nose-only air (control, Ba) or water pipe smoke (WPS, Bb) exposure, respectively. al, alveolus. Data are mean ± SEM (*n* = 6).

The number neutrophils per high power field (×100) in the lung of WPS-exposed mice was significantly increased compared to control group (*P* < 0.05; Fig.2A). The number of lymphocytes in WPS-exposed group was also increased compared to air-exposed group (*P* < 0.001; Fig.2B).

### Total protein, LDH, and endothelin in BAL fluid

**Figure 3:**
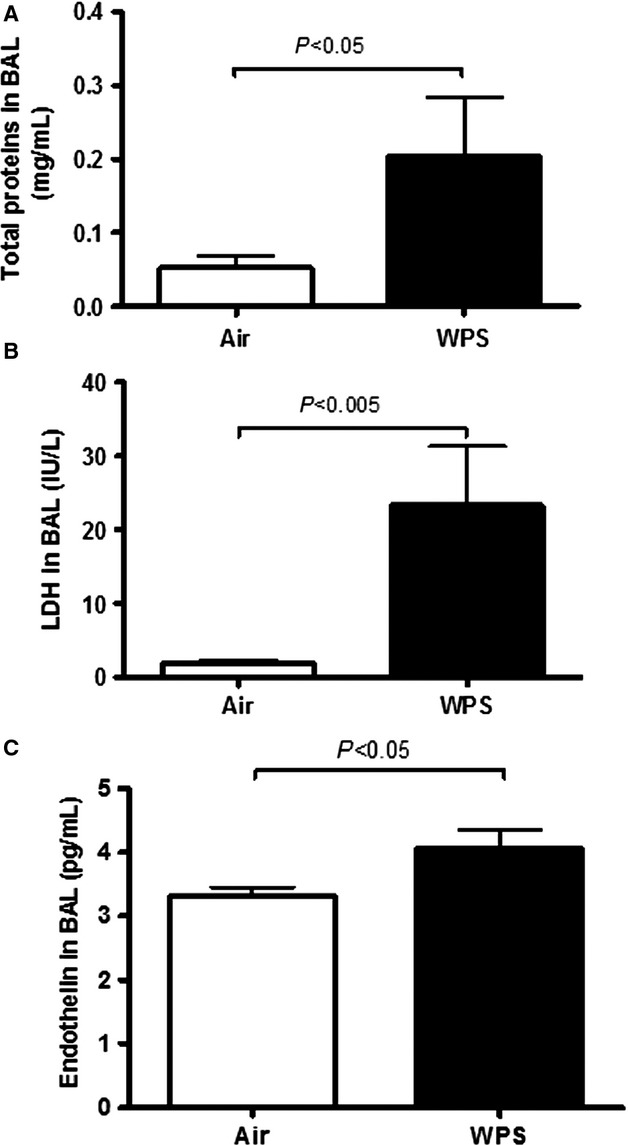
Total proteins concentration (A), lactate dehydrogenase (LDH) activity (B), and endothelin concentration (C) in bronchoalveolar lavage (BAL) fluid following subacute exposure to air (control) or water pipe smoking (WPS). Data are mean ± SEM (*n* = 8).

The total protein, used as a marker of epithelial permeability, was significantly increased (*P* < 0.05) following subacute nose-only WPS exposure compared with air-exposed mice (Fig.3A). The activity of LDH, a marker of cytotoxicity, was also significantly augmented (*P* < 0.005) in WPS group compared to control group (Fig.3B). Similarly, endothelin concentration was also enhanced (*P* < 0.05) after WPS exposure (Fig.3C).

### TNFα and IL-6 in lung tissue

**Figure 4:**
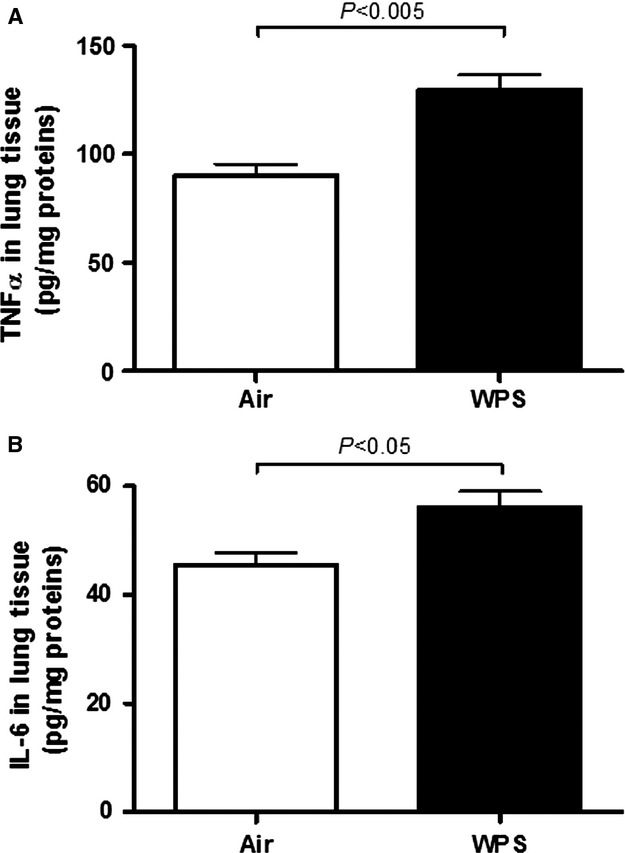
Tumor necrosis factor α (TNFα, A) and interleukin-6 (IL-6, B) in lung tissue following subacute exposure to air (control) or water pipe smoking (WPS). Data are mean ± SEM (*n* = 8).

TNFα concentration in lung homogenates was significantly increased (*P* < 0.005) following nose-only WPS exposure compared to air-exposed mice (Fig.4A). Likewise, compared with air-exposed group, IL-6 concentration was significantly augmented (*P* < 0.05) following nose-only WPS exposure (Fig.4B).

### ROS, LPO, and GSH concentrations and catalase and SOD activities in lung tissue

**Figure 5:**
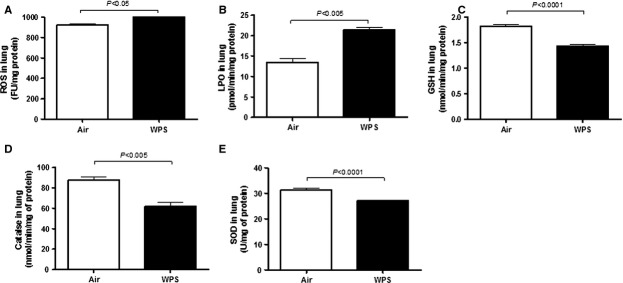
Reactive oxygen species (ROS, A), lipid peroxidation (LPO, B), reduced glutathione (GSH, C), catalase (D), and superoxide dismutase (E) in lung tissues following subacute exposure to air (control) or water pipe smoking (WPS). Data are mean ± SEM (*n* = 8).

Figure5 illustrates the effects of subacute WPS exposure on markers of oxidative stress including ROS, LPO, GSH, catalase, and SOD. After exposure to WPS, both ROS and LPO concentrations in the lung increased significantly compared with air exposure (Fig.5A and B). On the other hand, the concentration of GSH (Fig.5C) and the activities of catalase (Fig.5D) and SOD (Fig.5E) were significantly decreased in mice exposed to WPS for 5 days compared with those exposed to air for the same duration. The last result indicates the occurrence of the oxidative stress that caused the decrease of the antioxidant levels in the lungs.

### Airway hyper-reactivity to methacholine

**Figure 6:**
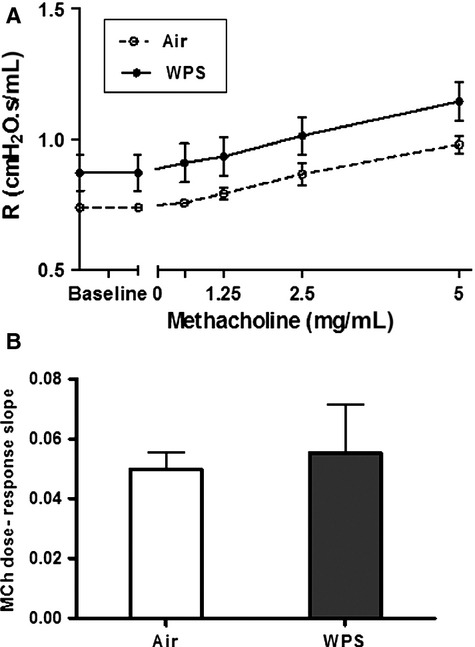
Airway responsiveness to methacholine (MCh). The airway resistance (*R*), after increasing concentrations of MCh (0–5 mg/mL), was measured via the forced oscillation technique (FlexiVent) following subacute exposure to air (control) or water pipe smoking (WPS). Dose–response relationship of total respiratory system resistance to increasing doses of MCh (A). From the resistance MCh dose–response curve in (A), an index of airway responsiveness was calculated as the slope of the linear regression using 0–5 mg/mL concentrations (B). Data are mean ± SEM (*n *=* *8).

Figure6 shows airway resistance, measured by the forced oscillations technique after increasing concentrations of methacholine (0–5 mg/mL), following nose-only exposure to air or WPS. From the resistance methacholine dose–response curve, an index of airway responsiveness was calculated as the slope of the linear regression using 0–5 mg/mL concentration. The latter showed no significant increase of airway resistance in WPS–exposed mice compared with air-exposed ones (Fig.6B), although a trend in increase in airway resistance baseline has been observed in WPS group.

## Discussion

This study provides evidence that subacute (5 days) nose-only exposure to WPS caused early pulmonary inflammation evidenced by infiltration of neutrophils and lymphocytes, increased epithelial permeability, and concentrations of proinflammatory cytokines such as TNFα and IL-6 in lung tissue. The ROS and LPO concentrations also increased in lung tissue. The antioxidant enzymes levels including GSH, SOD, and catalase decreased by WPS. However, at the time point investigated, the airway resistance was not affected by WPS.

Although extensive experimental work has been done on CS and its effects on pulmonary function and inflammation, there is very limited number of studies on the biological effect of WPS. We have recently demonstrated that 1-month nose-only WPS exposure cause lung inflammation and oxidative stress, and increased airway resistance in mice (Nemmar et al. 2013c). In this study, we continued to apply the same relevant type of exposure system (i.e., nose-only), and we wanted to assess whether and to what extent does subacute exposure to WPS affect lung function, inflammatory response, and oxidative stress. Studying the early pulmonary events related to WPS exposure can give more specific information and reflect on the initial changes in the lung that will eventually lead to chronic pulmonary effects of WPS.

Our data show that subacute exposure to WPS triggered lung inflammation substantiated by interstitial infiltration of inflammatory cells consisting in neutrophils and lymphocytes with shorter bronchiolar epithelium in lung sections obtained from mice exposed to WPS. We also found a significant increase in LDH activity, suggestive of cytolysis, and proteins in BAL fluid, reflecting increased epithelial permeability. Intratracheal instillations of aqueous cigarette smoke extract in rats (Qamar and Sultana 2008) or nanoparticles in hamsters (Nemmar et al. 2003) have been reported to cause increase of LDH and total protein in BAL fluid. We found an increase in endothelin concentration in BAL fluid. We have reported similar observation in mice exposed to cigarette smoke (4 days, nine cigarettes/day; Nemmar et al. 2012a). Endothelin has been reported to cause neutrophils recruitment in the lung and the pharmacological blockage of this inflammatory mediator has been shown to mitigate lipopolysaccharide and CS-induced lung inflammation (Bhavsar et al. 2008). Furthermore, we found a significant increase in TNFα and IL-6 concentrations in lung tissues of WPS-exposed mice compared to those exposed to air. An increase of neutrophil and lymphocyte numbers along with IL-6 and TNFα was observed in BAL fluid of mice exposed to WPS in whole-body exposure system, 1 h daily for 7 days (Khabour et al. 2012). We have recently reported that a 1-month exposure to WPS causes pulmonary infiltration of neutrophils and lymphocytes, and increase of both IL-6 and TNFα in BAL fluid (Nemmar et al. 2013c). It has also been demonstrated that TNFα in mice drives the majority of cigarette smoke-related emphysema (Churg et al. 2004). Using immunohistological techniques, it has been shown that there is a higher epithelial expression of TNF-α, IL-1β, and IL-6, as well as an activation of NF-κB and activator protein-1/mitogen-activated protein kinase signaling pathways in the respiratory tract of smoking patients, compared with the normal ciliated epithelium of nonsmoking patients (Herfs et al. 2012).

Oxidative stress describes the consequences of an imbalance between oxidant–antioxidant systems, with the outcome being favorable to oxidants (Birben et al. 2012). Several studies have reported higher levels of oxidative stress in cigarette smokers. The last effect was ascribed to the high concentration of ROS in cigarette smoke and the increased ROS production by infiltrating inflammatory cells (such as neutrophils) in the airways of both subacute and chronic smokers (Birben et al. 2012). Furthermore, excess CS-induced ROS that cannot be neutralized by endogenous antioxidants will ultimately result in oxidative stress-induced lung damage (Birben et al. 2012). To further evaluate the subacute effect of nose-only WPS on the lung, we have measured ROS and LPO in the lungs. Our data show that WPS exposure causes a significant increase in ROS and LPO concentrations in lung tissue. We have recently reported an increase in lipid peroxidation in lung tissues at the end of 1-month exposure to WPS (Nemmar et al. 2013c). With respect to CS, it has been demonstrated that short-term exposure induced an increase of ROS and lipid peroxidation (Nemmar et al. 2012a). In addition, in this study, we have measured the concentration of GSH, a free radical scavenger and two major antioxidant enzymes, namely, SOD and catalase. The catalase enzyme catalyzes the removal of H_2_O_2_ to stable O_2_ and the SOD enzyme is highly efficient in dismutating O_2_^−^ to H_2_O_2_ (Birben et al. 2012). Our data show a significant decrease in GSH concentration and the activities of catalase and SOD, suggesting a consumption of antioxidants during the breakdown of free radicals. Similar findings were observed in lung tissue of mice exposed to WPS for 1 month (Nemmar et al. 2013c). However, an increase of GPx and SOD in lung has been reported in mice exposed for 7 days in a whole-body exposure system (Khabour et al. 2012). Similarly, Valenca et al. (Valenca et al. 2008) found an increase in lung SOD, catalase, and GPx in mice exposed to CS for 5-days, using a whole-body exposure system. These findings suggest that the increase in oxidative stress was accompanied by increased antioxidant capacity, indicating that whole-body exposure to WPS and CS could trigger adaptive responses that counterbalance the potentially damaging activity of oxygen radicals and limiting further oxidant-mediated lung inflammation (Lykens et al. 1992). In our nose-only exposure system, the fact that we observed a decrease in antioxidant levels indicate their consumption during the breakdown of free radicals, and suggest that the exposure system may play a role in the effect of WPS or CS on oxidative stress. Additional studies using standardized condition of exposure to WPS, and comparing whole-body versus nose-only exposure systems are required to address this point.

Clinical studies reported impairment of pulmonary function following acute exposure to WPS (Hakim et al. 2011; Bentur et al. 2014). A significant decrease in the FEV_1_ (Raad et al. 2011), respiratory rate, PEFR, and FEF_25–75%_ have been observed in WPS group compared with nonsmokers (Hakim et al. 2011). Experimentally, the assessment of lung and airway mechanics with forced oscillation technique provides precise information, and physiological variables can be measured accurately (Vanoirbeek et al. 2010; De Vleeschauwer et al. 2011). We have recently demonstrated that 1-month exposure to WPS caused significant and dose-dependent increase of airway resistance (Nemmar et al. 2013c). In this study, we found no significant increase of airway resistance in WPS-exposed mice compared with air-exposed ones, although a trend in the increase of baseline airway resistance was seen in WPS group. This finding suggests that the inflammation and oxidative stress observed after subacute exposure to WPS did result in an immediate small but not significant increase of airway resistance. However, if WPS exposure is repeated over a longer period of time (e.g., 1-month exposure), it will ultimately lead to a significant airway hyper-reactivity and alteration of pulmonary function, as recently reported by us (Nemmar et al. 2013c).

In conclusion, our data demonstrate that subacute (5-day) nose-only exposure to WPS caused pulmonary inflammation and oxidative stress without affecting pulmonary function suggesting that inflammation and oxidative stress are early markers of WPS exposure that precedes airway dysfunction. Our data provide specific information on the initial changes in the lung after subacute exposure to WPS, which may constitute the underlying causal chain of reactions leading to the ultimate long-term pulmonary effects. Our study provides biological plausibility for the injurious effects of WPS after a relatively short duration of exposure, and support interventions to control the worldwide spread of WPS, particularly among the young generation.
